# Influence of Crystallite Size on the Magnetic Order in Semiconducting ZnCr_2_Se_4_ Nanoparticles

**DOI:** 10.3390/ma12233947

**Published:** 2019-11-28

**Authors:** Ewa Malicka, Małgorzata Karolus, Tadeusz Groń, Adrian Gudwański, Andrzej Ślebarski, Jerzy Goraus, Monika Oboz, Bogdan Sawicki, Joanna Panek

**Affiliations:** 1 Institute of Chemistry, University of Silesia, 40-007 Katowice, Poland; ewa.malicka@us.edu.pl (E.M.); agudwanski@gmail.com (A.G.); 2 Institute of Material Science, University of Silesia, 40-007 Katowice, Poland; joanna.panek@us.edu.pl; 3 Institute of Physics, University of Silesia, 40-007 Katowice, Poland; tadeusz.gron@us.edu.pl (T.G.); andrzej.slebarski@us.edu.pl (A.Ś.); jerzy.goraus@us.edu.pl (J.G.); monika.oboz@us.edu.pl (M.O.); bogdan.sawicki@us.edu.pl (B.S.)

**Keywords:** nanoparticles, semiconductors, antiferromagnetic, spin-glass, specific heat

## Abstract

Structural, electrical, magnetic, and specific heat measurements were carried out on ZnCr_2_Se_4_ single crystal and on nanocrystals obtained from the milling of this single crystal after 1, 3, and 5 h, whose crystallite sizes were 25.2, 2.5, and 2 nm, respectively. For this purpose, the high-energy ball-milling method was used. The above studies showed that all samples have a spinel structure, and are *p*-type semiconductors with less milling time and *n*-type with a higher one. In turn, the decrease in crystallite size caused a change in the magnetic order, from antiferromagnetic for bulk material and nanocrystals after 1 and 3 h of milling to spin-glass with the freezing temperature *T*_f_ = 20 K for the sample after 5 h of milling. The spin-glass behavior for this sample was derived from a broad peak of dc magnetic susceptibility, a splitting of the zero-field-cooling and field-cooling susceptibilities, and from the shift of *T*_f_ towards the higher frequency of the ac susceptibility curves. A spectacular result for this sample is also the lack of a peak on the specific heat curve, suggesting a disappearance of the structural transition that is observed for the bulk single crystal.

## 1. Introduction

The search for new materials for applications in thermoelectric devices is becoming more and more popular. Seleno-spinels are interesting compounds useful for this purpose [1,2,3], possessing a large cubic cell with an edge of 10 Å, a large ion radius of selenium (198 pm [4]), and strong covalence [5]. Pure ZnCr_2_Se_4_ in both mono and polycrystalline form is a *p*-type semiconductor, has a helical antiferromagnetic structure (AFM) below the Néel temperature *T*_N_ = 20 K, and a strong ferromagnetic component (FM), as evidenced by the high positive Curie–Weiss temperature (θ) of 115 K [6,7]. At *T*_N_ temperature, there is a structural phase transition from cubic *Fd*-3*m* to tetragonal symmetry *I*4_1_/*amd* with slight contraction along the *c* axis [8,9]. ZnCr_2_Se_4_ is a normal spinel in which zinc ions occupy tetrahedral sites and chromium ions occupy octahedral ones. Replacing one element with another or adding another element can strongly affect the physico-chemical properties of the matrix [10,11,12,13,14].

Recently, a CuCr_2_S_4_ spinel was obtained from pure elements Cu, Cr, and S by mechanical alloying (MA), whose crystallite size was on the order of 2.8 nm [15]. This method showed that, instead of the classic metallic ferromagnet, we received an antiferromagnetic semiconductor also with a spinel structure. Moreover, spin frustration for further Cr–Cr ion coordination spheres was observed [15]. 

In the present work, we obtained ZnCr_2_Se_4_ nanopowders, by the high-energy ball-milling method, from the milling of the ZnCr_2_Se_4_ single crystal after 1, 3, and 5 h. Structural, magnetic, electronic transport, and specific heat studies were carried out on the single crystal and nanopowder samples. We expect these studies to show that obtaining nanoscale ZnCr_2_Se_4_ crystallites will significantly change their physical properties.

## 2. Materials and Methods 

The nanosized crystallites of ZnCr_2_Se_4_ spinel were made with the use of the high-energy ball-milling (HEBM) method. The starting materials for the HEBM method were single crystalline samples to avoid impurity phases, whose lack was confirmed by powder X-ray diffraction studies. 

Single crystals of ZnCr_2_Se_4_ were grown by chemical vapor transport technique with the anhydrous chromium chloride as the carrier agent. A stoichiometric mixture of the selenide ZnSe and transporting agent CrCl_3_ were enclosed in evacuated (~10^−3^ Pa) and sealed quartz ampoules (a length of about 200 mm and an inner diameter of 20 mm). The ampoules were heated in a two-zone gradient furnace for 3 weeks. The optimal temperatures at the crystallization and melting zones were between 1123 and 1223 K, respectively. The right choice of transport conditions resulted in obtaining good quality octahedral crystals with surface types (111).

Milling was carried out in PULVERISETTE 7 premium line Planetary Ball Mill (Fritsch, Germany) at room temperature with stainless steel vessels and balls (5 mm). Single crystals were milled up to 5 h at the speed of 500 rpm with a 10:1 ball-to-crystals ratio in dry conditions. Milling of single crystals was carried out under a protective argon (Ar) atmosphere. 

The powders obtained after 1, 3, and 5 h of milling were characterized by X-ray diffraction technique (Empyrean, PANalytical, Almelo, Netherlands) and scanning electron microscopy (SEM).

The X-ray diffraction experiment was performed with the Empyrean PANalytical Diffractometer with copper radiation (λCu_Kα_ = 1.5418 Å) and PIXcel counter. The phase analyses were performed with the ICDD PDF4+ 2017 crystallographic database. The determination of unit cell parameters, crystallite sizes, and lattice strain values, as well as qualitative phase analysis, were refined by the use of the High Score Plus PANalytical software (HSP version 3.0, PANalytical, Almelo, Netherlands) based on the Williamson–Hall theory [16] and Rietveld refinement [17,18]. The scheme of analyses, conditions, and accuracy of methods were presented in detail in, among other works [19,20].

The morphology of the powders was studied by the use of a scanning electron microscope (SEM) (Jeol JSM-6480) (JEOL Ltd, Tokyo, Japan).

The electrical conductivity *σ*(T) was measured in the 74–410 K temperature range using the dc method and KEITHLEY 6517B Electrometer/High Resistance Meter (Keithley Instruments, LLC, Solon, OH, USA). The thermoelectric power *S*(T) was measured in the temperature range of 100–400 K by a Seebeck Effect Measurement System (MMR Technologies, Inc., San Jose, CA, USA). The static dc magnetic susceptibility was measured in both the zero-field cooled (ZFC) and the field-cooled (FC) modes in the magnetic field *H*_dc_ = 1 kOe. The calculations of the effective magnetic moment, and the method of preparation of powder samples for measurements of electrical conductivity and thermopower, are described in detail in [15,21,22,23]. Magnetization isotherms were measured at 2, 10, 20, 40, 60, and 300 K in applied external fields up to 70 kOe. Magnetization and dc magnetic susceptibility were carried out in the temperature range of 2–300 K using a Quantum Design MPMS-XL-7AC SQUID magnetometer (Quantum Design, San Diego, CA, USA). For ac magnetic susceptibility in the frequency range 300 Hz–10 kHz, and with specific heat measurements, a Quantum Design Physical Properties Measurement System (QD-PPMS) (Quantum Design, San Diego, CA, USA) was used. The superexchange integrals for the first two coordination spheres *J*_1_ and *J*_2_ were calculated with the aid of the Holland and Brown equations: *J*_1_ = (−9*T*_N_ + θ)/60 and *J*_2_ = (3*T*_N_ + θ)/120 [24]. The exchange constant *J*_SG_ of the spin-glass system was estimated using the random energy model [25,26,27] by the expression: *J*_SG_ = T_f_⋅(4⋅ln2)^1/2^ [25].

## 3. Results and Discussion

### 3.1. Structural Characteristics

**Figure 1:**
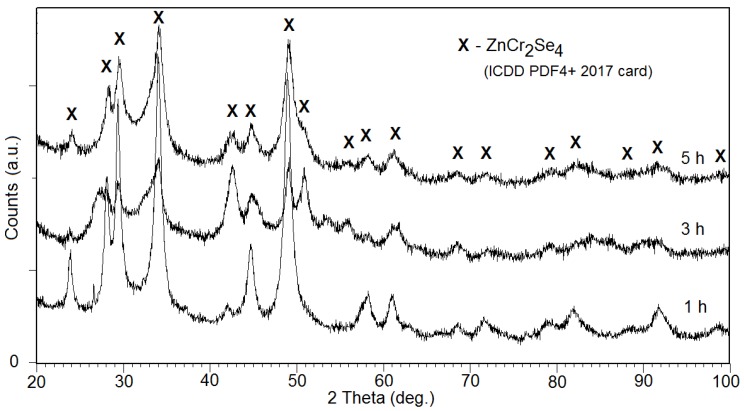
The X-ray diffraction patterns of spinel ZnCr_2_Se_4_ (ICDD PDF4+ 2017: 03-065-0689 card) after 1, 3, and 5 h of milling.

X-ray diffraction patterns analysis (Figure 1) indicated the presence of spinel ZnCr_2_Se_4_ (*Fd*-3*m*) in all samples (ICDD PDF4+ 2017: 03-065-0689). Structural analysis carried out by the use of the Rietveld procedure allowed the determination of lattice parameters of nanocrystalline spinel after a specified milling time and its size of crystallites and lattice strains (Table 1). During the milling process, the size of spinel ZnCr_2_Se_4_ crystallites decreased from 25.2 nm (for 1 h), then 2.5 nm (for 1 h) to 2 nm (for 5 h) (Table 1). The significant change of lattice strain (τ) value obtained after 5 h of milling and visible change of peak intensities (e.g., 2θ = 28, 32 diffraction line) might indicate the unit cell deformation and appearance of texture in the material. The refinement parameters obtained during the analysis process reached acceptable [17,18,19,20] levels of: R_exp_ = 5.1–10.7%; R_p_ = 5.8–12.1%; R_wp_ = 7.5–10.1%; and goodness-of-fit (S) = 1.7–1.9.

**Figure 2:**
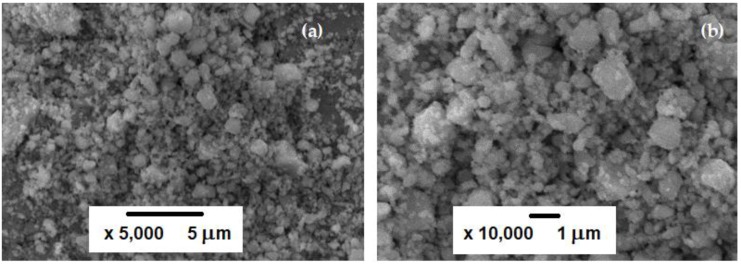
SEM images of ZnCr_2_Se_4_ powder after 5 h of milling magnified (**a**) 5,000 times and (**b**) 10,000 times.

SEM images of the investigated ZnCr_2_S_4_ powder after milling for 5 h, presented in Figure 2, showed morphology typical of materials subjected to high-energy ball-milling. Repeated processes occurring during milling lead to the formation of small particles. The formation of smaller or larger agglomerates, consisting of submicro-sized or nanosized particles, was an effect of crushing and subsequent agglomeration by cold welding, fracturing, and re-welding. The particles visible in the SEM images represent grains and their agglomerates of various sizes, from 0.1 to 2 μm.

### 3.2. Electrical Properties

**Figure 3:**
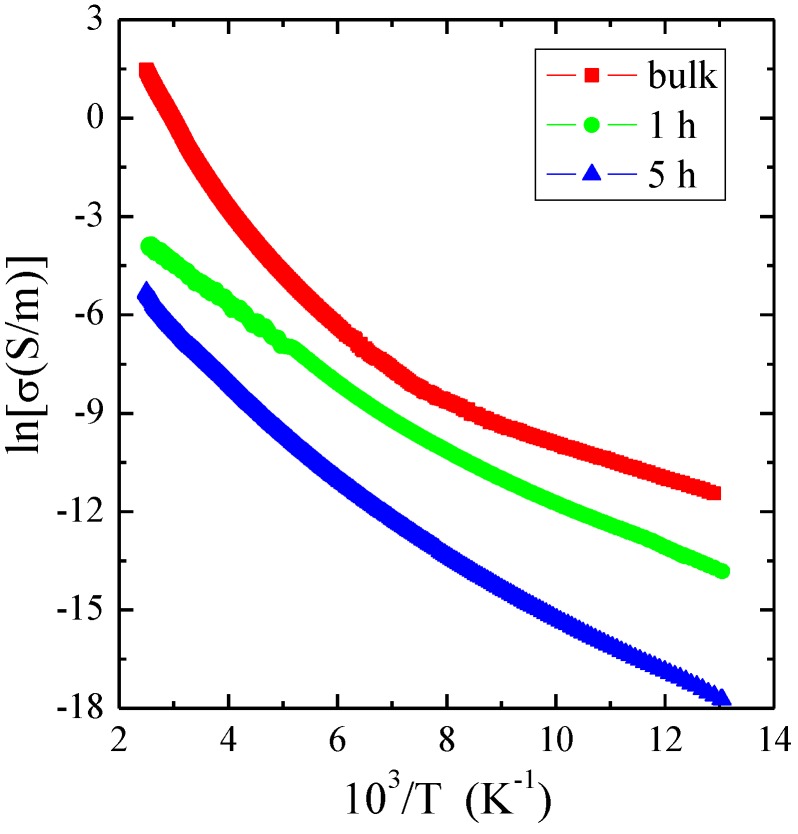
Electrical conductivity (ln*σ*) vs. reciprocal temperature 10^3^/*T* measured for ZnCr_2_Se_4_ single crystal and nanocrystals after 1 and 5 h of milling.

The electrical conductivity (σ) measurements of single crystal and nanocrystals after 1 and 5 h of milling, displayed in Figure 3, showed semiconducting behavior. As the crystallite size decreased, the electrical conductivity decreased in the measured temperature range. The electrical conductivity of all samples was thermally activated, and activation energy (*E*_A_) slightly increased in the extrinsic region from 0.05 eV (for bulk), then 0.06 eV (for 1 h) to 0.07 eV (for 5 h). In the intrinsic region, *E*_A_ changed from 0.22 eV (for bulk), then 0.10 eV (for 1 h) to 0.14 eV (for 5 h). A much smaller *E*_A_ of nanoparticles in the intrinsic region compared to a bulk material may be the result of the formation of agglomerates composed of submicro-sized or nanosized particles, visible on the SEM image. 

**Figure 4:**
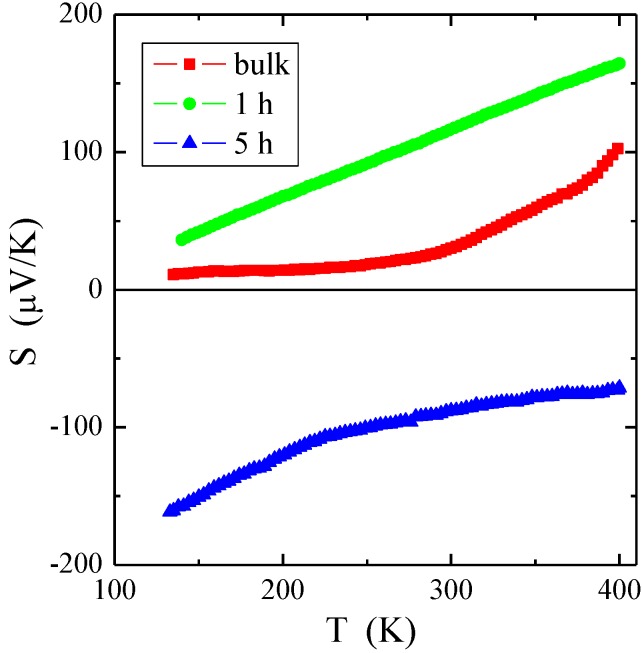
Thermoelectric power *S* vs. temperature *T* measured for ZnCr_2_Se_4_ single crystal and nanocrystals after 1 and 5 h of milling.

Temperature measurements of thermoelectric power (Figure 4) showed the dominance of holes in the bulk material (well-known in the literature [7,28]) as well as in the sample after one hour of milling, and the dominance of electrons in the sample after five hours of milling. In the latter, the electron conductivity may have been due to the appearance of donor (anion) vacancies and structural defects during the formation of small particles as a result of crushing. In the materials of the spinel structure, strong ionic bonds existed between cations and anions. The appearance of a non-zero value of the electrical conductivity in spinels might have been caused by both the existence of mixed valence bands and ion vacancies [29]. In the ZnCr_2_Se_4_ spinel, which is a *p*-type semiconductor, chromium ions were in the 3*d t*_2g_ ground state and did not form a band of mixed valence. Therefore, after a longer milling time, there was a predominance of anion vacancies over cationic ones, which was manifested by a change in the type of conduction from *p* to *n*.

### 3.3. Magnetic Properties

**Figure 5:**
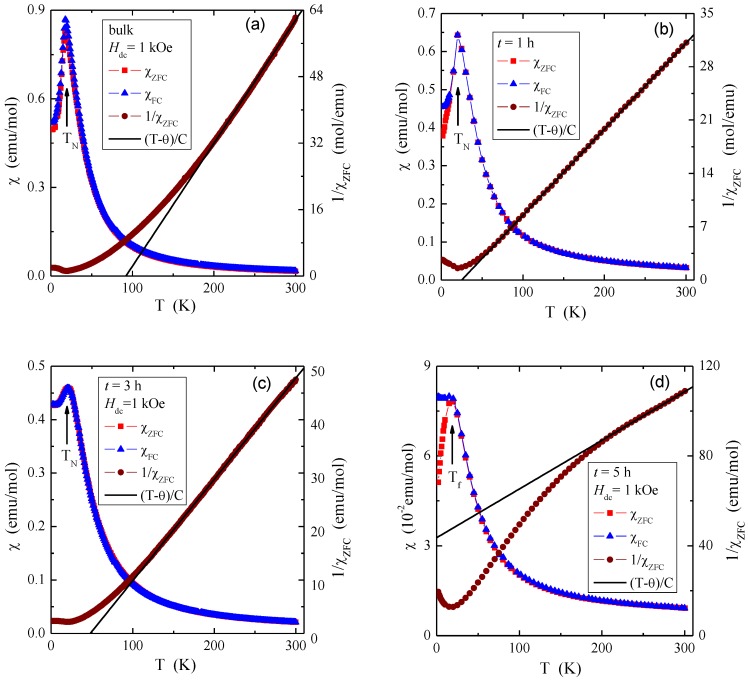
Zero-field-cooled (ZFC) and field-cooled (FC) magnetic susceptibility *χ* vs. temperature *T* at *H*_dc_ = 1 kOe for ZnCr_2_Se_4_ single crystal (**a**) and nanocrystals after 1 h (**b**), 3 h (**c**), and 5 h (**d**) of milling. *T*_N_ and *T*_f_ are the Néel and freezing temperatures, respectively.

**Figure 6:**
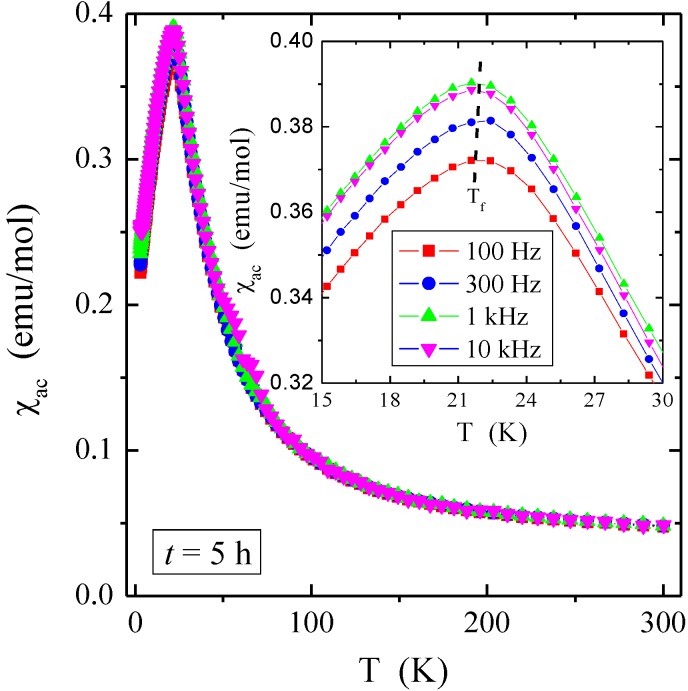
In-phase component of ac magnetic susceptibility *χ*_ac_ vs. temperature *T* at *H*_ac_ = 2 Oe recorded at different frequencies and after 5 h of milling. Inset: *χ*_ac_*(T)* magnification in the range up to 30 K.

The temperature dependence of dc magnetic susceptibility, both ZFC (χ_ZFC_) and FC (χ_FC_), showed AFM order for bulk material and nanocrystals after 1 and 3 h of milling with the Néel temperatures of 18, 20, and 20 K, respectively (Figure 5, Table 2). At the same time, the positive value of the paramagnetic Curie–Weiss temperature (θ), characteristic of the short-range FM interactions, varied from 92 (for bulk), then 25 (for 1 h) to 48 K (for 3 h). The nanocrystal after 5 h of milling showed unexpectedly ferrimagnetic (FIM) long-range interactions visible in the deviation of the curve of the inverse magnetic susceptibility (1/χ_ZFC_) from its linear part, and AFM short-range interactions visible in the negative value of paramagnetic Curie–Weiss temperature of θ = −201 K (Table 2), as well as the spin-glass-like (SG) behavior, evidenced by the splitting of the ZFC and FC magnetic susceptibility below the freezing temperature of *T*_f_ = 20 K (Figure 5). The confirmation of the existence of the SG state was also the shift of the ac magnetic susceptibility peak towards the higher frequencies (Figure 6) that is described by the Vogel–Fulchel law [30]. However, this shift was not exactly monotonous with frequency, hence the Vogel–Fulchel law cannot be fit reliably. Usually, the ZnCr_2_Se_4_ single crystals described in the literature have a positive and high Curie–Weiss temperature in the range of θ = 90–115 K [6,7,9], just like the single crystal in this study. 

The reduction in crystallite size strongly affected both the value and the sign of this temperature (Table 2). All nanocrystals had significantly higher values of the effective magnetic moment, compared to the theoretical value of μ_eff_ = 5.477 μ_B_/f.u., in which the Cr^3+^ ions were in the 3*d t*_2g_ high-spin configuration, and the experimental values of 5.197 and 5.714 μ_B_/f.u. for the single crystal under study, and taken from [9], respectively (Table 2). It may indicate that, in the studied spinel nanoparticles, magnetic dipoles or magnetic cluster structure had formed.

The crystallite size also affected the magnetic interactions described by the superexchange integrals (Table 2). For single crystal and nanocrystals after 1 and 3 h of milling, the superexchange integral for the first coordination sphere (*J*_1_) is negative and becomes more negative for nanocrystals. In turn, the superexchange integral for the second coordination sphere (*J*_2_) is positive and becomes less positive for nanocrystals. In this case, the *J*_1_ integral represents the antiferromagnetic long-range interactions, while the *J*_2_ integral represents the short-range ferromagnetic ones. As a consequence, for nanocrystals after 5 h of milling, i.e., for the sample with the smallest crystallite size (Table 1), the spin-glass state whose exchange integral is large, i.e., *J*_SG_ = 33.3 K (Table 2), was observed. For comparison, *J*_SG_ = 73.3 K for spin-glass-like behavior in single-crystalline Cu_0.44_In_0.48_Cr_1.95_Se_4_ spinel was found [31].

**Figure 7:**
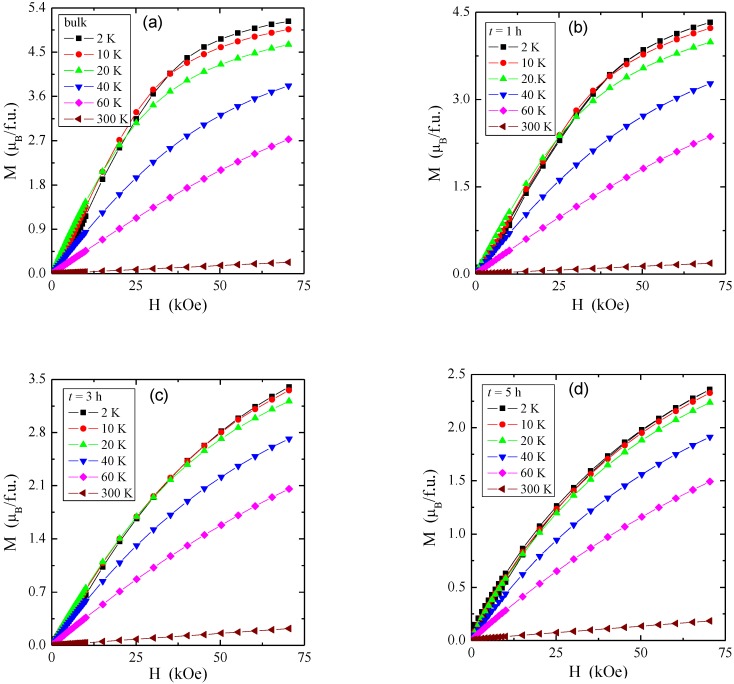
Magnetization *M* vs. magnetic field *H* at 2, 10, 20, 40, 60, and 300 K kOe for ZnCr_2_Se_4_ single crystal (**a**) and nanocrystals after 1 h (**b**), 3 h (**c**), and 5 h (**d**) of milling.

The magnetic isotherms of the single crystal and the nanocrystals after 1, 3, and 5 h of milling are displayed in Figure 7. With the reduction of crystallite size, magnetization at 2 K, and in the magnetic field of 75, kOe decreases from 5.125 µ_B_/f.u. (for bulk) to 2.358 µ_B_/f.u. (for 5 h), and the magnetic isotherms move away from the state of saturation. On magnetic isotherms at 2 K and in the magnetic field of 10 kOe, the inflection points are clearly visible, which are characteristic of the first critical field associated with the transition from the helical phase to the conical phase (metamagnetic threshold) for single crystal and nanocrystals after 1 and 3 h of milling. As the milling time increases, we observe the shift of the first critical field towards smaller fields and its disappearance for a 5 h sample. A similar shift is observed for M–H hysteresis (not visible in Figure 7), as well as, e.g., in NiO nanocrystals [32]. The second critical field associated with the transition from the magnetic cone structure to the ferromagnetic phase was not observed for any of the studied samples in the magnetic field up to 75 kOe. It is usually perceived in a magnetic field of 65 kOe [9] when the magnetic isotherm reaches saturation.

### 3.4. Specific Heat

**Figure 8:**
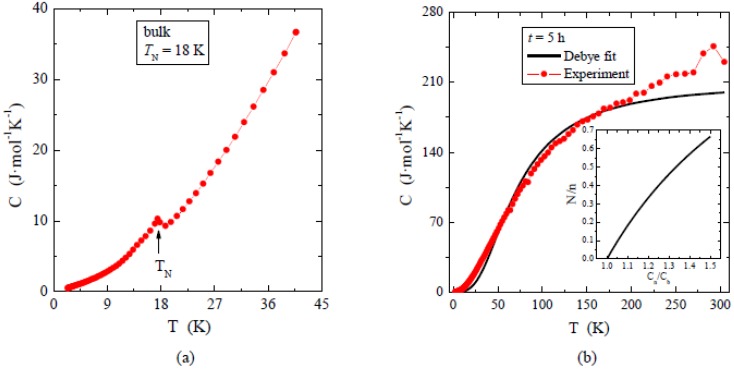
Specific heat *C* vs. temperature *T* in zero-magnetic field measured for the single crystal (**a**) and for nanocrystals after 5 h of milling (**b**). This sum contains electronic, antiferromagnetic, and lattice contribution. In the right panel, the solid line represents the Debye fit. In the inset we show: *N*/*n* vs. *C*_n_/*C*_b_, where *N* is the number of atoms on the surface, *n* is the total number of atoms, *C*_n_ is the specific heat measured for nanoparticles, and *C*_b_ is the specific heat measured for the bulk solid lattice.

Figure 8 shows the specific heat measured up to 40 K for the single crystal (8a) and up to 300 K for nanocrystal after 5 h of milling (8b). The specific heat *C (T)* measured for the single crystal shows at the temperature *T*_N_ λ-shape peak characteristic of the second-type paramagnetic-antiferromagnetic order. In the temperature range T > *T*_N_ the specific heat is usually expresses by phonon contribution *C(T)* = *βT*^3^. However, in the case of monocrystalline ZnCr_2_Se_4_, the *C (T)* ~ *T*^3^ behavior is limited only to narrow temperature range *T*_N_ < *T* < 28 K, because of presence of spin fluctuations [11] that modify the lattice contribution to specific heat. In this narrow temperature range *β* = 0.00045 J/mol K^4^. For *N* = 7 atoms in formula unit, *β* = *N(12/5)π^4^R*θ_D_ gives the Debye temperature θ_D_ ≈ 311 K. For the nanocrystalline material, we also fitted the Debye model to the C (T) data (as is shown in Figure 8b) in the wide temperature range, assuming that the spin fluctuations are removed in nano-grained material. The obtained fit parameters were: Debye temperature θ_D_ = 290 K and the number of atoms *n*_D_ = 8.38. The number of atoms is significantly larger than the expected 7 for the ZnCr_2_Se_4_ formula unit. The simple explanation of this phenomenon can be provided on the basis of the model published in [30], where the authors have shown that, for the nanoparticles, the specific heat is larger than for the bulk solid. The inset presents the fraction of the number of atoms on the surface *N* to the total number of atoms *n*, versus the ratio of specific heat measured for nanoparticles *C*_n_ to the specific heat measured for the bulk solid lattice *C*_b_. In our case, such an estimation shows that 32% of atoms are on the surface, which clearly confirms that our sample was milled down to a few nm in size. In Figure 8a, a clear peak at *T*_N_ in the specific heat curve of a single crystal is observed, but no peak is visible in specific heat for the nanocrystal after 5 h of milling (Figure 8b), which suggests that, for such small nanoparticles, no long range magnetic order was present. There is a spread in the size of nanoparticles. Despite the fact that most of them are in the nm range, a small fraction can be larger and exhibit magnetic ordering, which is visible in the ac magnetic susceptibility.

## 4. Conclusions

The nanocrystals were obtained using the high-energy ball-milling method from the ZnCr_2_Se_4_ single crystal after 1, 3, and 5 h of milling, whose crystallite sizes were 25.2, 2.5, and 2 nm, respectively. Structural, electrical, magnetic, and specific heat measurements showed that all samples had a spinel structure, were *p*-type semiconductors with less milling time, and *n*-type with a higher one. With a decrease in crystallite size, the magnetic order changed from antiferromagnetic for bulk material and nanocrystals after 1 and 3 h of milling to the spin-glass state for the nanocrystal after 5 h of milling. The spin-glass-like behavior was derived from a broad peak of dc magnetic susceptibility, a splitting of the zero-field-cooling and field-cooling susceptibilities, and from the shift of the freezing temperature towards the higher frequency of the ac susceptibility curves. The spin-glass-like system was also confirmed by the large exchange constant of 33.3 K, which was additionally accompanied by the lack of a peak on the specific heat curve, suggesting a disappearance of the structural transition.

In summary, decreasing the spinel monocrystal crystallite resulted in a change in magnetic order from AFM to SG, an increase in AFM long-range interactions, and weakening of FM short-range ones that finally led to a strong competition of FIM and AFM interactions in the spin-glass sample with the smallest crystallite size.

## Figures and Tables

**Table 1:** Structural parameters of ZnCr_2_Se_4_ nanospinel.

*t* (h)	*a* (Å)	*d* (nm)	τ (%)
1	10.5064(5)	25.(2)	0.8
3	10.4856(4)	2.(5)	1.1
5	10.4758(3)	2.(0)	1.3

*t* is the milling time, *a* is the unit cell parameter, *d* is the crystallite size, and τ is the lattice strain. For comparison, the theoretical unit cell parameter of ZnCr_2_Se_4_ bulk spinel (a = 10.4970 Å) was taken from the ICDD PDF4+ 2017: 03-065-0689 card.

**Table 2:** Magnetic parameters of ZnCr_2_Se_4_ nanospinels.

*t* (h)	*d* (nm)	*C* (emu⋅K/mol)	*T*_N_ (K)	θ (K)	µ_eff_ (µ_B_/f.u.)	*M*_(2K)_ (µ_B_/f.u.)	*J*_1_ (K)	*J*_2_ (K)	Ref.
-	bulk	4.082	21	90	5.714	6.0	−1.65	1.28	[9]
-	bulk	3.377	18	92	5.197	5.125	−1.17	1.22	this paper
1	25	8.841	20	25	8.409	4.328	−2.58	0.71	this paper
3	2.5	5.273	20	48	6.494	3.401	−2.20	0.90	this paper
5	2	4.611	*T*_f_ = 20 K	−201	6.073	2.358	*J*_SG_ = 33.3 K		this paper

*t* is the milling time; *d* is the crystallite size; *C* is the Curie constant; *T*_N_, *T*_f_, and θ are the Néel, freezing, and Curie–Weiss temperatures, respectively; μ_eff_ is the effective magnetic moment; *M* is the magnetization at 2 K; *J*_1_ and *J*_2_ are the superexchange integrals for the first two coordination spheres; and *J*_SG_ is the exchange constant of the spin-glass system. For comparison, magnetic parameters of ZnCr_2_Se_4_ bulk single crystal from the literature [9] were taken.
